# Storage and diffusion of CO_2_ in covalent organic frameworks—A neural network-based molecular dynamics simulation approach

**DOI:** 10.3389/fchem.2023.1100210

**Published:** 2023-03-09

**Authors:** Bernhard M. Kriesche, Laura E. Kronenberg, Felix R. S. Purtscher, Thomas S. Hofer

**Affiliations:** ^1^ Institute of General, Inorganic and Theoretical Chemistry, Center for Chemistry and Biomedicine, University of Innsbruck, Innsbruck, Austria

**Keywords:** covalent organic framework, carbon dioxide, diffusion, neural network potential, molecular dynamics, CO2 storage, minimum distance distribution functions, pore size distribution

## Abstract

As a consequence of the accelerated climate change, solutions to capture, store and potentially activate carbon dioxide received increased interest in recent years. Herein, it is demonstrated, that the neural network potential ANI-2x is able to describe nanoporous organic materials at approx. density functional theory accuracy and force field cost, using the example of the recently published two- and three-dimensional covalent organic frameworks HEX-COF1 and 3D-HNU5 and their interaction with CO_2_ guest molecules. Along with the investigation of the diffusion behaviour, a wide range of properties of interest is analyzed, such as the structure, pore size distribution and host-guest distribution functions. The workflow developed herein facilitates the estimation of the maximum CO_2_ adsorption capacity and is easily generalizable to other systems. Additionally, this work illustrates, that minimum distance distribution functions can be a highly useful tool in understanding the nature of interactions in host-gas systems at the atomic level.

## 1 Introduction

In the past decade the impact of global climate change has become increasingly visible, thereby evolving this pressing issue from a purely academic discussion towards intensified activities to implement climate protection involving all levels of society. However, the recent reports of the Intergovernmental Panel on Climate Change (IPCC) Pörtner et al. (2022) have concluded, that the effects of global climate change are occurring on shorter time scales than initially projected Tollefson (2022). While a number of factors contribute to this development, the increase in greenhouse gas concentrations resulting from different human activities represent a key component accelerating global warming. In particular the increased carbon dioxide (CO_2_) content in the atmosphere is proven to be a main contributing factor next to methane (CH_4_) emission Lashof and Ahuja (1990) and the formulation of sustainable strategies to reduce CO_2_ emission have become the focus of increasing research activities Boot-Handford et al. (2014). Among those, capture, fixation and potential activation of carbon dioxide in suitable absorption media Elhenawy et al. (2020) such as metal- and covalent organic frameworks (MOFs, COFs) are largely regarded as one of the most promising routes Ding et al. (2019); Ozdemir et al. (2019). This is due to the fact that these highly porous compounds display very large storage capacities of gaseous guest molecules, that can be further enhanced *via* suitable substitutions Yuan et al. (2021); An et al. (2019). In particular, azine-linked COFs such as HEX-COF1 Alahakoon et al. (2016) and 3D-HNU-5 Guan et al. (2019) have been reported to display an exceptionally high affinity towards carbon dioxide. In fact, the above-mentioned compounds rank among the best performing storage media for CO_2_ surpassing the capacities of natural compounds such as zeolites. Geng et al. (2020).

Owing to the fact that these supramolecular compounds oftentimes have complex unit cells, the theoretical treatment of these systems is typically associated with a large computational demand, especially when applying quantum chemical levels of theory such as density functional theory (DFT) Sholl and Steckel (2009); Koch and Holthausen (2002). The latter is especially true when aiming at the diffusive properties of guest molecules inside supramolecular structures. In this case comparably long molecular dynamics simulations are required, so that the associated diffusion coefficient can be evaluated in the long-time limit. While classical molecular mechanical (MM) potentials provide a low-cost alternative to quantum mechanical (QM) calculations, the inherent consideration of complex interactions such as many-body and polarization effects makes QM approaches the preferred level of theory to study the associated host-guest interactions.

With the increasing success of machine-learning approaches, effectively influencing every discipline of modern sciences, neural network potential (NNP) models Kocer et al. (2022); Behler (2011) emerged as a further alternative to the established MM and QM methods Kulichenko et al. (2021). In particular, the ANI (Accurate NeurAl networK engINe for Molecular Energies, ANAKIN-ME) NNP Smith et al. (2017), focused on the treatment of organic molecular systems, displays a number of promising features that can be exploited to study the properties of gaseous guest molecules in COF systems. Initially designed for the treatment of molecules composed of C, H, N and O atoms based on an extensive database of molecular structures treated at density functional theory (DFT) level, the ANI NNP is capable of replicating the structural description of the DFT training set at a computational cost comparable to simple molecular force fields. Smith et al. (2017) Moreover, it was shown that this approach provides an accurate description of molecules being significantly larger than those included in the training set, which could be further improved in the second iteration, *i.e.*, the ANI-2 NNP Devereux et al. (2020).

While the treatment of condensed solid-state structures is typically more challenging than calculations of isolated molecules in vacuum or implicit solvation, the large porosity of COF structures can be expected to mimic a vacuum environment to a great extent. Provided, that the chosen NNP is sufficiently accurate to model the solid-state system, a versatile simulation approach to investigate the storage and diffusion properties of gas@COF systems could be formulated.

**FIGURE 1:**
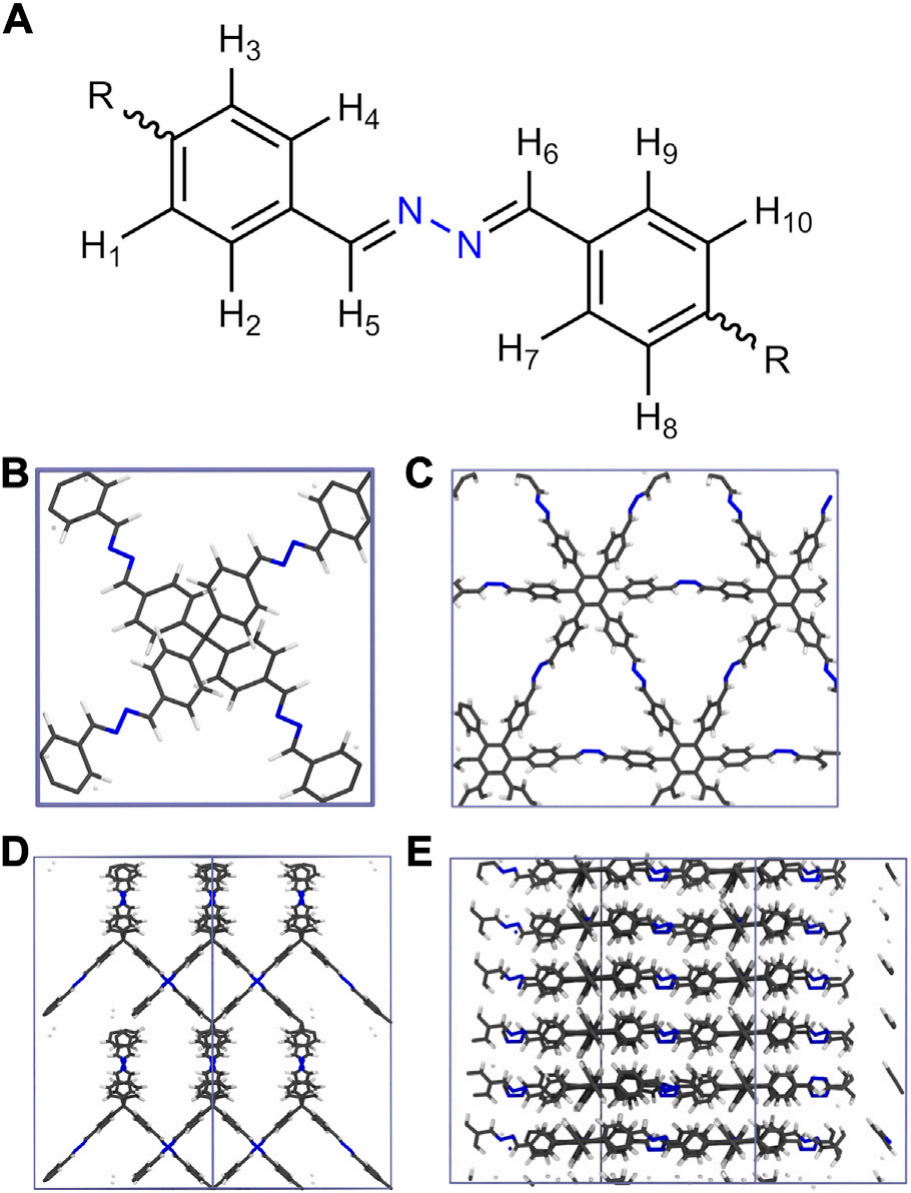
**(A)** Azine linking unit comprising both HEX-COF1 and 3D-HNU5 as well as the numbering scheme used to identify unique hydrogen atoms (*vide infra*), **(B–E)** Depiction of the unit cells of 3D-HNU5 and HEX-COF1 employed in the MD simulations in top view **(B, C)** and side view **(D, E)**, respectively.

In this work the ANI-2 NNP has been combined with a suitable molecular dynamics (MD) framework to enable the treatment of HEX-COF1 Alahakoon et al. (2016) and 3D-HNU-5 Guan et al. (2019), two COF systems that share the same linking unit shown in Figure 1A. HEX-COF1 has a 2-dimensional structure, forming layers that stack on top of each other, 3D-HNU5 on the other hand is a 3-dimensional COF in which the linkers are oriented in a tetrahedral geometry, forming a 2-fold interpenetrated diamond topology Figure 1B–E. These COF systems have been shown to possess distinctly dissimilar CO_2_ adsorption capacities in experiments Alahakoon et al. (2016); Guan et al. (2019). This difference in the absorption properties between these highly similar COF systems (i.e., identical linking units) was one of the reasons to select these particular systems for the current study. This enables the investigation of the different CO_2_ storage characteristics based on an identical structural motif in a 2D- and 3D-COF environment. Prior to loading the COFs with increasing amounts of CO_2_ molecules at different state points, the performance of the ANI-2 NNP in the description of the pristine solids has been critically assessed.

## 2 Methodology

### 2.1 The ANI-2x neural network potential

Artificial neural network (ANN) approaches are generally regarded as universal function approximators. Hornik et al. (1989) Their utilization within the field of chemistry, comprises *inter alia* the training of a neural network model to reproduce reference energies from either experiment or more commonly quantum chemical calculations. While the construction of such a neural network potential (NNP) is oftentimes computationally very demanding, especially if the generation of *ab initio* or DFT reference data is taken into account, the prediction of properties from a built model is similar to the cost of classic MM force-fields. Smith et al. (2017) This approach of reproducing reference data is also conceptually reminiscent of classic force fields, albeit with the important distinction, that it has been more fruitful to create accurate general purpose ML potentials, that are trained on a large set of chemically diverse structures. This enhanced transferability is in parts due to the adoption of a so-called atomistic perspective, Schütt et al. (2019) which assumes, that extensive properties (*e.g.*, energy) can be expressed as the sum of single atom contributions. To create suitable inputs for the NNP, the Cartesian coordinates are mapped to atomic environment vectors (AEV) which reflect the local atomic environment in a unique and roto-translationally invariant way. Behler (2011) Consequently, ANNs are superior in extrapolating to yet unseen data, especially to larger systems, than they were trained on. This opens the possibility to create universal NNPs, that yield chemically accurate predictions over a wide range of chemical space and do not need to be reparametrized for narrow groups of molecules, as is often the case with classical force fields.

One of the most successful freely available, open-source NNPs to date is ANI (ANAKIN-ME, Accurate NeurAl networK engINe for Molecular Energies), which was initially trained on molecules containing the elements C, H, N and O Smith et al. (2017) but was later extended to include S as well as the halogens F and Cl in the second generation ANI-2x. Devereux et al. (2020).

### 2.2 Setup of the simulation cells

For 3D-HNU5, the unit cell information reported by Guan and coworkers Guan et al. (2019) was employed as a starting point, to generate an orthorhomibic 2 × 2 × 2 supercell, with optimized cell parameters *a* = *b* = 31.62Å, *c* = 36.94Å. The latter correspond to a unit cell with dimensions *a* = *b* = 15.81Å, *c* = 18.47Å, which compares favorably to the experimental values of *a* = *b* = 15.143488Å, *c* = 19.967805Å.

In case of HEX-COF1, the source publication Alahakoon et al. (2016) did not include any structural data, but information about the associated lattice parameters. Based on this information, the unit cell could be constructed from scratch using GaussView 6.0.16 Dennington et al. (2019) to replicate the provided structural depictions Alahakoon et al. (2016). Next, a 2 × 2 × 6 supercell containing 8 channels with the optimized cell parameters being *a* = 34.12Å, *b* = 29.80Å, *c* = 31.64Å was generated.

### 2.3 Preliminary assessment of the host-gas interaction

To gauge the general ability of ANI-2x to adequately reflect the molecular species and their governing interactions, a series of preliminary calculations was performed. In particular, the interaction energy between both 3D-HNU5 (unit cell) and HEX-COF1(1 × 1 × 3 supercell) and a single CO_2_ molecule was calculated with a wide array of computational methods in addition to ANI-2x. The list includes also increasingly successful density functional tight binding (DFTB) approaches in addition to different density functionals, most of which are many orders of magnitude more expensive than the ANI-2x framework. With each of the methods, the structure of the pristine COF, a single CO_2_ molecule, and the CO_2_@COF system was optimized and the interaction energy calculated according to Eq. (1).

$$E_{\mathrm{int}}=E_{COF+CO_{2}}-E_{\mathrm{COF}}-E_{{\text{CO}}_{2}}$$ (1)

Further it was ensured, that the system size is adequate to avoid artificial CO_2_—CO_2_ interactions across the periodic boundary (see Section 1.2, Supporting Information).

### 2.4 Simulation protocol

All conducted simulations were performed using the velocity-Verlet algorithm Leach (2001); Jensen (2017) to integrate the equations of motion in conjunction with the neural network potential (NNP) ANI-2x employed to execute the energy and force calculations, while applying periodic boundary conditions in all three spatial dimensions. In all simulations, bonds containing hydrogen atoms were constrained to the ensemble average determined from initial simulation trajectories using the SHAKE/RATTLE Ryckaert et al. (1977); Andersen (1983) algorithms. This enables an increase of the simulation time step to Δ*t* = 2.0 fs. The ensemble averages for each of the two systems were determined based on a 50 ps sampling run under *NPT* conditions, at 298.15 K and 1.013 bar. In both systems aromatic and olefinic C-H were set to a different target distance. The resulting average bond lengths along with their respective standard deviation^1^ are listed in Table 1.

**TABLE 1:** Ensemble average bond distances used to constrain bonds containing aromatic and olefinic hydrogen atoms for both 3D-HNU5 and HEX-COF1.

	C-H_*ole*_/Å	C-H_*ar*_/Å
3D-HNU5	1.100 162 9 (10)	1.090 149(3)
HEX-COF1	1.103 027 7(8)	1.090 839(4)

For thermostatization the Berendsen weak-coupling thermostat Berendsen et al. (1984) with a relaxation time *τ* = 0.1 ps was employed. For pressure control, a Monte-Carlo (MC) based manostat Åqvist et al. (2004) was employed, where every 50 fs (= 25 MD steps) a trial Monte-Carlo volume change is performed. The maximum trial step size $\frac{\left|\Delta V_{max}\right|}{V}$ is initialized with 1e-4 and adjusted every 10 MC steps based on a target acceptance ratio set to 30%. Further, *NPT* simulations of both systems were calculated XY-isotropically, *i.e.*, the simulation cell lengths along the X- and Y-axis were only permitted to vary collectively, but independently of the cell length in Z-direction.

A sampling frequency of 25 MD steps (50 fs) was used for all sampling runs. For 3D-HNU5 Guan et al. (2019) and HEX-COF1 Alahakoon et al. (2016), the empty system was first equilibrated under *NVT* conditions at 298.15 K for 100 ps and subsequently under *NPT* conditions at 298.15 K and 1.013 bar for 500 ps, using a time step of 0.5 fs.

To investigate the carbon dioxide adsorption capabilities of both 3D-HNU5 and HEX-COF1, CO_2_ molecules were incrementally introduced into the pre-equilibrated host structure. Each of the generated systems with distinct composition (*cf.* Table 2) was studied at a wide range of temperatures, *i.e.* 223.15–348.15 K in 25 K increments, in the case of HEX-COF1 also 198.15 K was included (see Supplementary Table S1, Supporting Information).

**TABLE 2:** Simulated compositions of the gas@host systems considered in case of 3D-HNU5 and HEX-COF1.

	n (CO_2_)_total_	n (CO_2_)/pore	w% CO_2_
3D-HNU5	1	-	0.6
	2	-	1.3
	4	-	2.6
	8	-	5.2
	16	-	10.4
	24	-	15.6
	32	-	20.7
	64	-	41.5
	128	-	82.9
HEX-COF1	8	1	2.1
	16	2	4.2
	32	4	8.5
	64	8	17.0
	80	10	21.2
	96	12	25.5
	128	16	34.0

For 3D-HNU5, each combination of composition and temperature was first equilibrated at the target temperature for 0.5 ns, followed by 1 ns equilibration under a constant pressure of 1.013 bar. Finally, 3.5 ns sampling runs under *NPT* conditions were conducted.

For HEX-COF1 a different scheme was adopted, as under *NPT* conditions for temperatures *T* > 298.15 K the individual layers of the COF tend to move relative to each other within the XY-plane leading to a misalignment of the different layers. This is in line with the conjecture, that the missing face-to-face π stacking interaction of the phenyl groups in HEX-COF1 could result in reduced interlayer adhesion Alahakoon et al. (2016). To avoid this undesired behaviour, for each individual composition, the system was equilibrated for 100 ps under *NVT* conditions at 298.15 K, followed by 400 ps under *NPT* conditions at 1.013 bar. Subsequently, the system was heated to the different target temperatures and left to equilibrate for 0.5 ns under *NVT* conditions. Finally, 3.5 ns sampling runs under *NVT* conditions were conducted.

### 2.5 MDDF analysis

To achieve a better understanding of the interactions between the guest molecules and the host structure, minimum distance distribution functions Martínez and Shimizu (2017) (MDDFs) were calculated. MDDFs are a novel analysis method, similar to radial distribution functions (RDFs). In contrast to the latter, MDDFs do not describe the relative distribution between pre-selected atoms of two predefined molecular species, but rather the relative distribution of minimum distances between any atom of the considered molecular species. As such MDDFs more intuitively reflect actual molecular interactions between complex molecular species. In addition, these distribution functions can be naturally decomposed into contributions of individual atoms of either of the two molecular species, which permits a more detailed view on the atoms involved in interactions. To successfully apply the MDDF formalism, initially developed for solvent-solute systems containing complex solutes, such as biomolecules, to the solid-gaseous systems investigated in the paper at hand, the following approach is pursued: *i*) the COF is used as though it was the solvent; *ii*) the solute molecules’ role is taken by gas (CO_2_) molecules.

### 2.6 Pore size distribution

To gain further insights into the properties of the host material, the associated pore size distributions (PSDs) for HEX-COF1 and 3D-HNU5 were calculated using two different geometric methods. First, a Monte-Carlo type insertion method based on the work by Gelb and Gubbins (1998) as implemented in the software PoreBlazer Sarkisov et al. (2020) was applied. In addition, an approach based on Voronoi-Tesselation as provided by the Zeo++ Willems et al. (2012); Pinheiro et al. (2013) package was employed.

### 2.7 Diffusion analysis

As outlined above, the diffusion coefficient *D* of CO_2_ in accordance with the Einstein relation given in Eq. (2) was determined from the simulation trajectories, with *d* being the dimensionality of the system, **r**_0_ and **r**_*t*_ correspond to the positions of the centre of mass of a CO_2_ molecule at the time origin and time t, respectively.

$$D=\frac{1}{2d}\underset{t\to \infty }{\lim}\frac{\left\langle \|r_{t}-r_{0}{\|}^{2}\right\rangle }{t}$$ (2)

For the calculation of these auto-correlation functions the full 3.5 ns trajectory of a given composition at a given temperature was analyzed with a large running correlation length of 75 ps and a window gap of 50 fs. Further, for the three-dimensional 3D-HNU5 system the total diffusivity was determined, whereas for the layered two-dimensional HEX-COF1 only the diffusion along the columnar pore, *i.e.*, in z-direction, was analyzed, as the spatial confinement renders diffusion in the xy-plane insignificant. To ensure that the data used for the determination of the self-diffusivity corresponds to motion in the diffusive regime, the linear regression was fitted only to the last 100 data points (5 ps) of a correlation interval. For each composition, the resulting diffusion coefficients were then used to calculate the average activation energy of diffusion according to

$$\ln\left(D\right)=-\frac{E_{a}}{R}\frac{1}{T}+\ln\left(D_{0}\right)$$ (3)

with *R*, *E*_*a*_ and *D*_0_ being the molar gas constant, the associated average activation energy, and the pre-exponential factor, respectively. Finally, for both COF systems, the calculated activation energies were analyzed, as a function of composition.

## 3 Results and discussion

### 3.1 Space group of HEX-COF1

The publication introducing the HEX-COF1 Alahakoon et al. (2016) system reports, based on their results, that its structure belongs to the space group P6/m. In this structural model the linking phenyl groups are aligned perfectly perpendicular to the central benzene moiety. The validity of this structure seems questionable, considering the fact, that the biphenyl molecule representing the simplest system containing two linked benzene rings, is known to be tilted. Almenningen et al. (1985); Grein (2002) For further investigation, two conformers of hexaphenylbenzene were used as a model system, with the orientation of the attached phenyl groups relative to the central benzene ring being perfectly perpendicular and slightly tilted, respectively. Both structures were subject to geometry optimization, followed by the calculation of the vibrational frequencies using the B3LYP/6-31++G (d,p) level of theory (*cf.* Supplementary Figure S1, Supporting Information). The optimized structure with a 90° dihedral, mirroring HEX-COF1 in the P6/m space group, showed two imaginary vibrational frequencies, confirming the structure to be a (second-order) saddle point on the energy landscape rather than a minimum. The structure considering tilted phenyl groups, corresponding to HEX-COF1 in the P6 space group, converged to an average dihedral angle between the aromatic rings amounting to approx. 68.3° and showed no imaginary vibrational frequencies, confirming it to be an energetic minimum. Consequently, this finding suggests, that HEX-COF1 is also likely to exist in the lower symmetry P6 space group.

### 3.2 Preliminary assessment of the host-gas interaction

**FIGURE 2:**
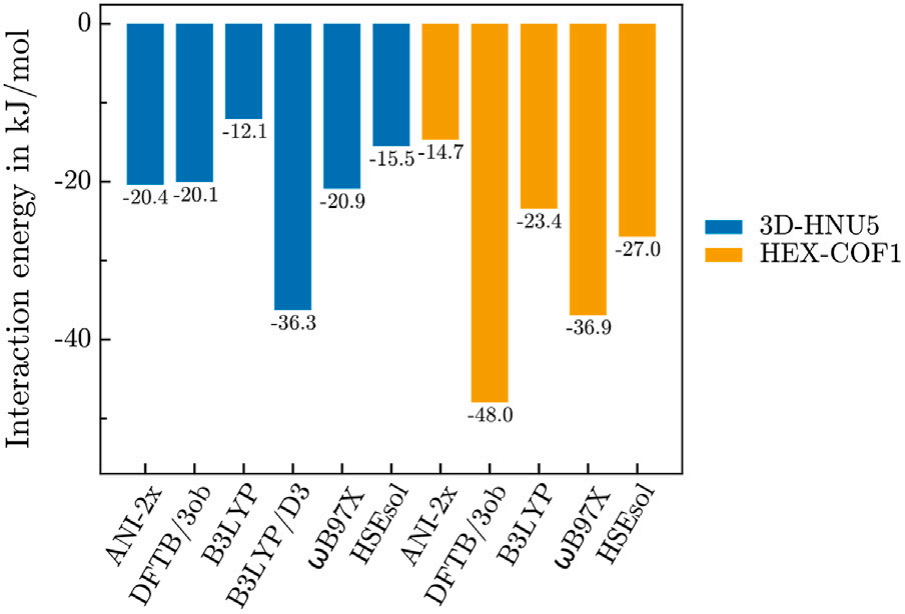
Interaction energies of 3D-HNU5 (unit cell) and HEX-COF1 (1 × 1 × 3 supercell) with a single CO_2_ molecule.

As mentioned above, the interaction energy between both HEX-COF1 and 3D-HNU5, and a single CO_2_ molecule was determined with an array of established DFT and DFTB methods, in order to warrant the suitability of applying the NNP ANI-2x to these systems prior to the execution of the MD simulations. The resulting data (see Figure 2) suggests, that for 3D-HNU5 the NNP results are well within the margin of uncertainty, observed for established computational methods, even more so considering the unprecedented computational efficiency. On the other hand, in case of HEX-COF1 ANI-2x finds a too low interaction energy. In contrast to ANI-2x, all reference methods predict a more favourable guest-host interaction in HEX-COF1 than in 3D-HNU5. Also, the standard deviation of the interaction energies produced by all reference methods (excluding B3LYP/D3) is approx. 3 times larger for HEX-COF1 than for 3D-HNU5. This suggests, that in the CO_2_@HEX-COF1 system a certain type of interaction is present, that the used reference methods incorporate to a varying degree, leading to a larger spread of the calculated interaction energies. Considering that the interaction site in HEX-COF1 and 3D-HNU5 contains the same azine-based linker units, the fact that ANI-2x displays the smallest deviation of 5.7 kJ ⋅ mol^-1^ appears quite promising nonetheless.

### 3.3 Structural deformation in 3D-HNU5

**FIGURE 3:**
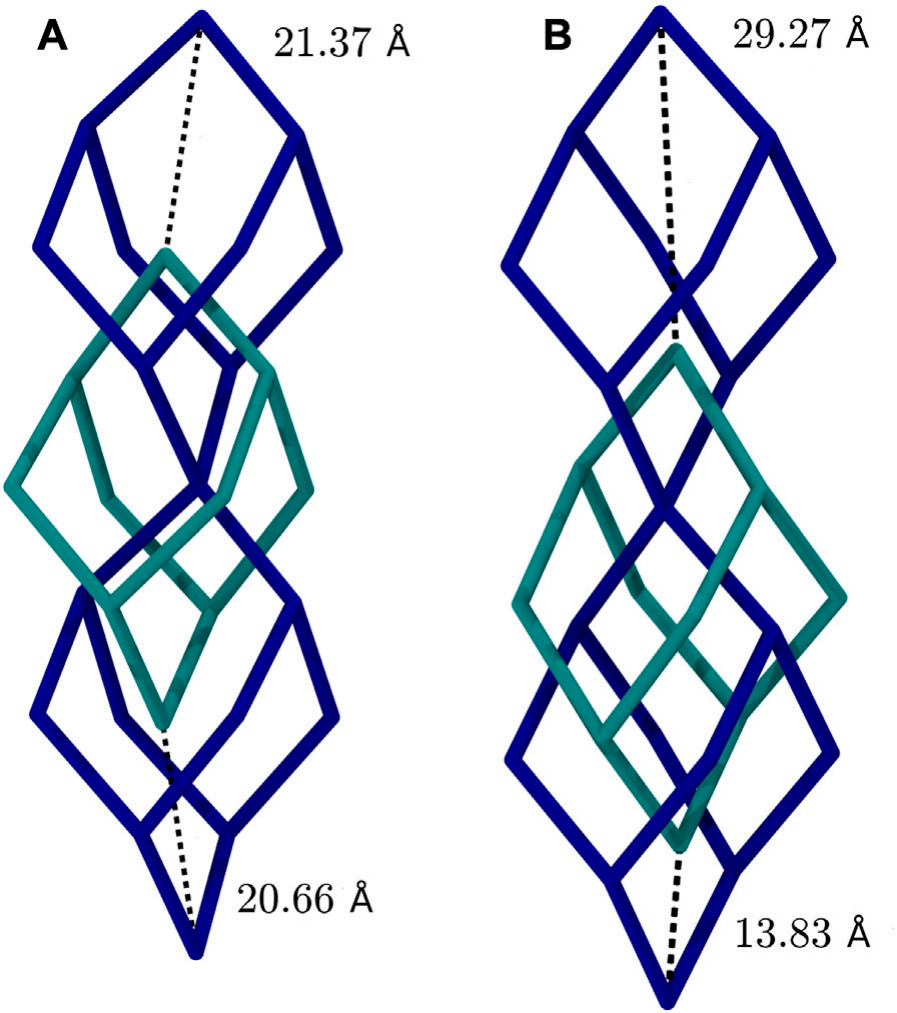
Comparison of 3D-HNU5 structures in two-fold interpenetrated diamond topology loaded with 10.4 w% CO_2_, **(A)** at 77.15 K, **(B)** at 273.15 K. Each bond represents an entire azine linking unit, that connects two central carbon atoms.

In case of 3D-HNU5 a distinct structural deformation upon exposition to external stimuli is observerd, in particular adsorption and temperature. This kind of behaviour has been reported for microporous materials with flexible molecular composition Gor et al. (2017), especially certain MOFs including MIL-53 Li et al. (2011); Coudert et al. (2013); Bakhshian and Sahimi (2018) and MIL-88 Coudert et al. (2013); Bakhshian and Sahimi (2018). To that effect, the structure of 3D-HNU5 is both highly flexible and very much dependent on *i*) the concentration of CO_2_ molecules present and *ii*) the applied simulation temperature. That is, for CO_2_ concentrations approaching and exceeding the experimentally determined maximum uptake capacity, the structure of the COF tends to be well-formed and highly symmetric with little variation in the pore diameter, while for lower concentrations of CO_2_ molecules, the framework exhibits a deformation, in which always two linkers congregate, and the two adjacent, formerly equally sized pores, form a smaller and a larger pore. For the CO_2_ concentrations, that mark the border between the symmetric and the deformed structural motif (*i.e.* 10.4, 15.6, 20.7 w%) the extent, to which the deformation is present, correlates with the applied simulation temperature, with lower temperatures favouring the symmetric and higher temperatures the deformed structure (see Figure 3).

### 3.4 MDDF analysis

**FIGURE 4:**
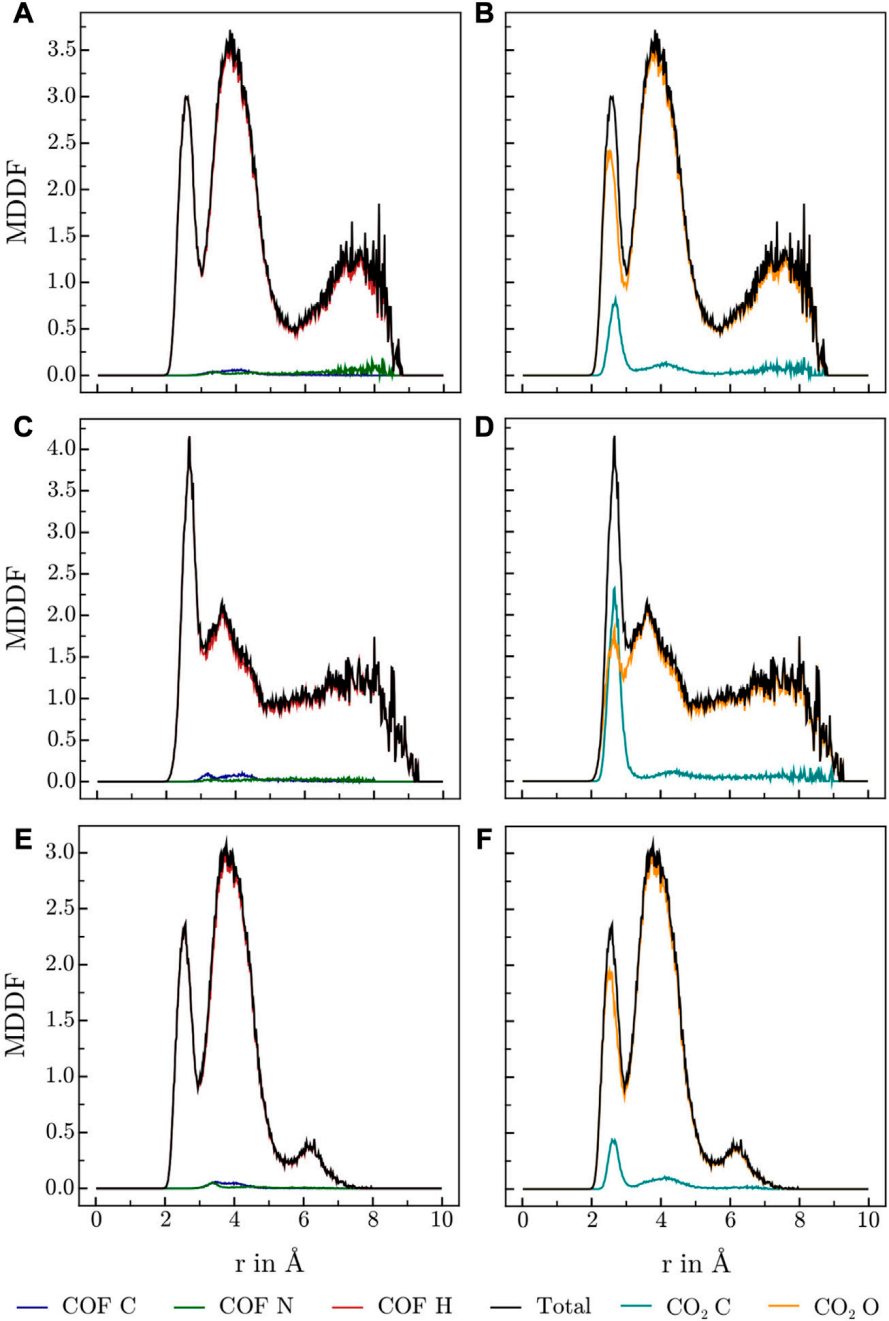
MDDFs obtained for 3D-HNU5 at various conditions, **(A, B)** 273.15 K and high CO_2_ concentrations (16 CO_2_), **(C, D)** 273.15 K and low CO_2_ concentrations (4 CO_2_), **(E, F)** 223.15 K and high CO_2_ concentrations (16 CO_2_), split into contributions of **(A, C, E)** COF atoms, and **(B, D, F)** CO_2_ atoms.

**FIGURE 5:**
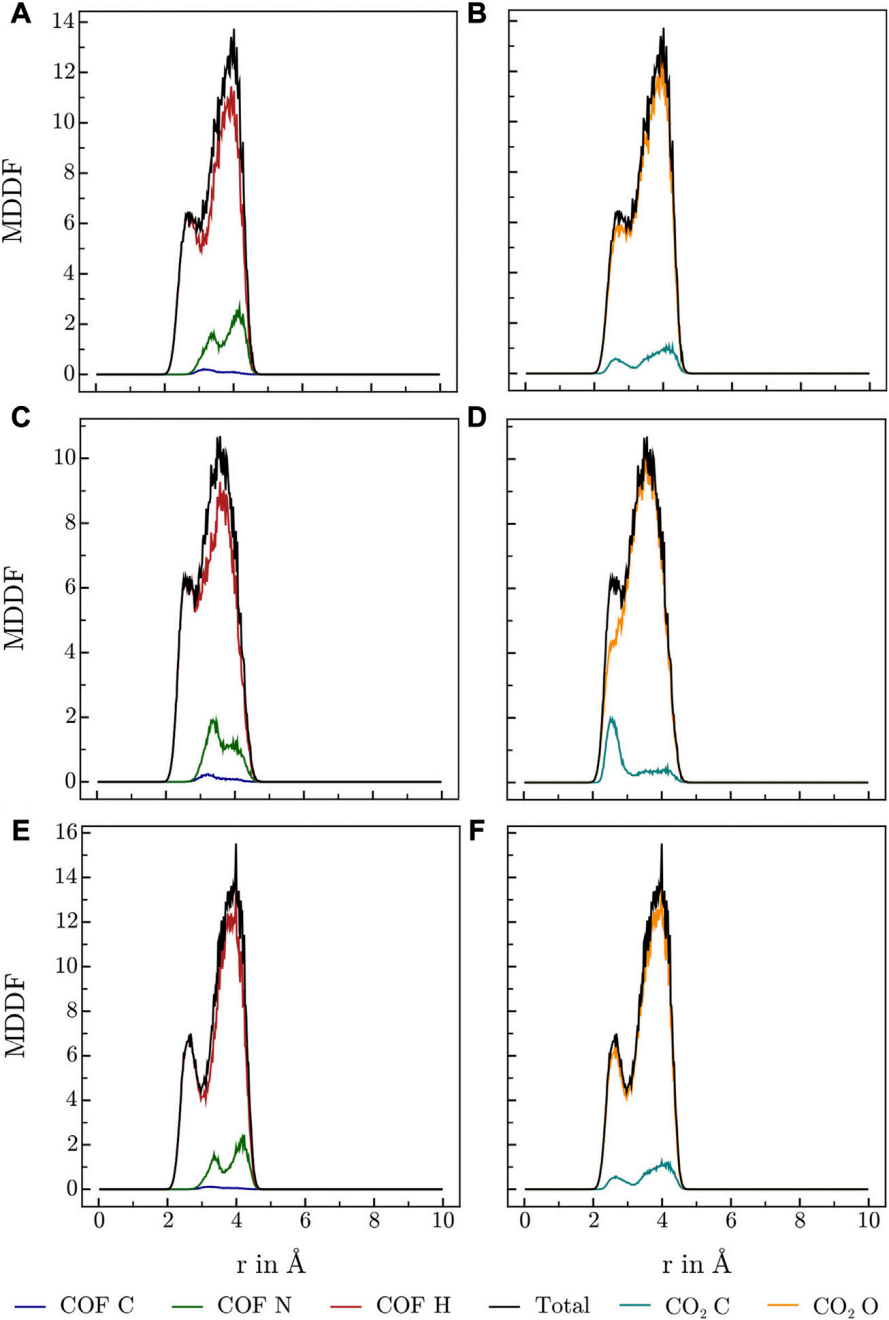
MDDFs obtained for HEX-COF1 at various conditions, **(A, B)** 273.15 K and high CO_2_ concentrations (8 CO_2_/pore), **(C, D)** 273.15 K and low CO_2_ concentrations (2 CO_2_/pore), **(E, F)** 223.15 K and high CO_2_ concentrations (8 CO_2_/pore), split into contributions of **(A,C,E)** COF atoms, and **(B, D, F)** CO_2_ atoms.

Various publications investigating CO_2_ adsorption in covalent Guan et al. (2019); Alahakoon et al. (2016); Pyles et al. (2016); Zhao et al. (2013); Huang et al. (2015) and metal organic frameworks Masoomi et al. (2014) suggest, that Lewis-basic hetero atoms in general and diazine linkers as found in HEX-COF1 and 3D-HNU5 in particular, along with a small pore diameter, increase the affinity and specificity for CO_2_ adsorption. To obtain further information on the host-gas interaction, the distribution of CO_2_ molecules inside each of the systems at hand was investigated by calculating the associated minimum distance distribution functions. By utilizing the decomposability of MDDFs into their atomic contributions, the involvement of the individual atoms of COF and CO_2_ in the guest-host interaction was investigated at multiple temperatures and CO_2_ concentration. The resulting MDDFs for 3D-HNU5 and HEX-COF1 are depicted in Figure 4, 5, respectively. Concerning the atoms of the COFs, the contributions of the N and C atoms are negligible for all investigated temperatures and CO_2_ concentration, for 3D-HNU5 almost vanishing, while the contributions of the hydrogens predominate. With regard to the individual atoms of the CO_2_ molecules, the contributions of the oxygen atoms account for the bigger part of the intermolecular interaction. The contributions of the carbon atoms are generally very small except for 3D-HNU5 at 273.15 K and low CO_2_ concentration, where the carbon atoms of CO_2_ account for a non-negligible, albeit overall minor contribution. In summary, the calculated minimum distances show, that for both systems the main guest-host interactions are found between hydrogen atoms of the COF structure and oxygen atoms of the individual CO_2_ molecules.

As this finding is in stark contrast to the above-mentioned sentiment that interactions with the azine groups are prevalent, a more detailed analysis was performed. Specifically, the hydrogen contribution to the MDDF was further split into groups of equivalent hydrogens, yielding a contribution for every topologically unique hydrogen of the azine linker. Figure 1A illustrates the numbering scheme used within this article to identify topologically unique hydrogens of the azine linker.

**FIGURE 6:**
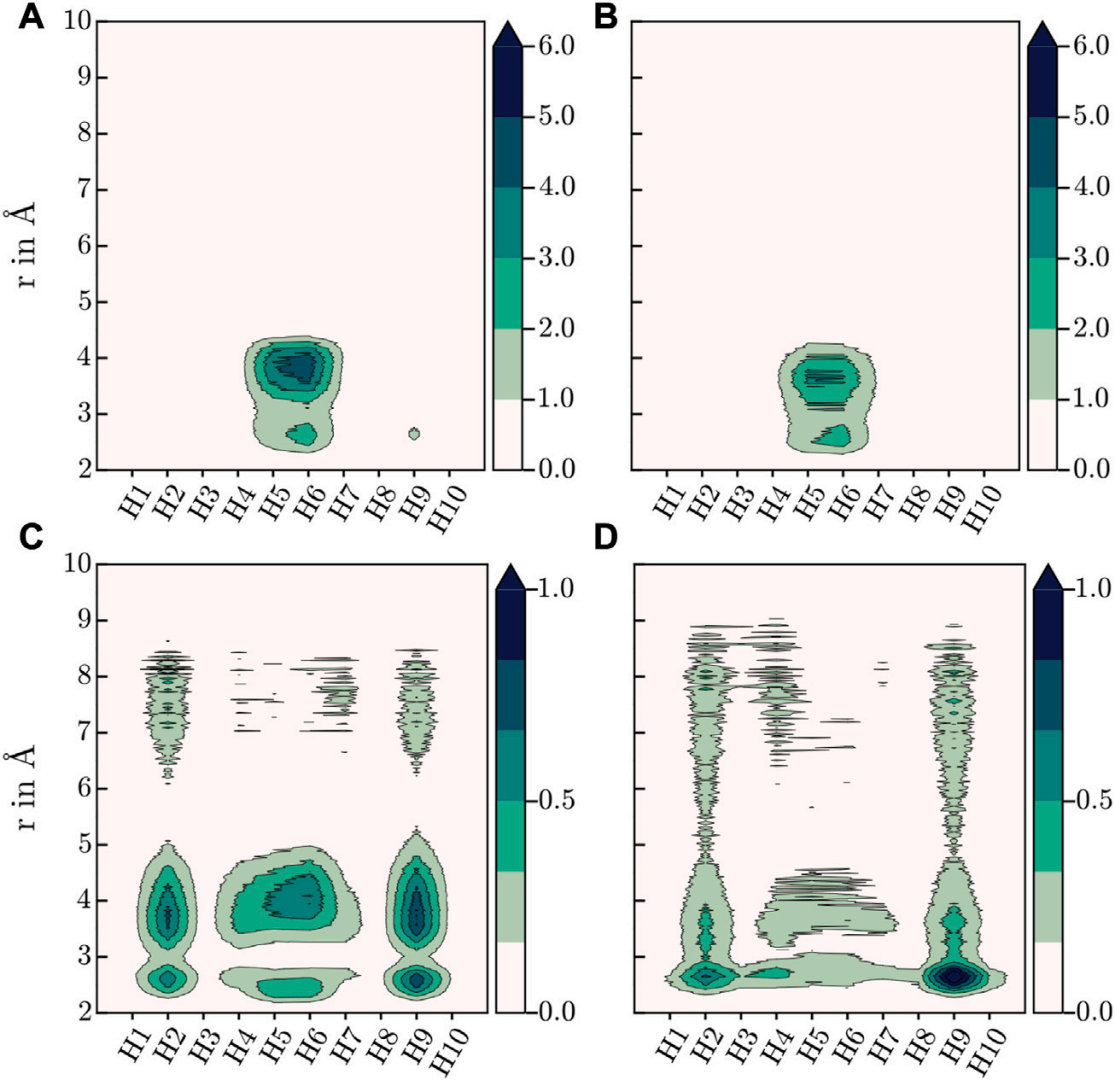
Contour plots of H-contributions to the total MDDF at 273.15 K, **(A)** HEX-COF1 with 8 CO_2_/pore, **(B)** HEX-COF1 with 2 CO_2_/pore, **(C)** 3D-HNU5 with 16 CO_2_, **(D)** 3D-HNU5 with 4 CO_2_ (see Figure 1A for labelling of the azine linker hydrogens).

The resulting contributions to the total MDDF of the individual H atoms are shown in contour plots in Figure 6. For HEX-COF1 this approach identified, that the most significant contributions stem from the olefinic hydrogens next to the azine group (H_5_ and H_6_ in Figure 1A). Consequently, this finding does not contradict the initial assumption, that the azine group plays an important part in the intermolecular interaction. Rather this result fortifies the assumption, as it stands to reason, that the introduced effect of the azine group is not mediated *via* direct interaction but rather indirectly *via* polarization of the associated methine groups.

In case of 3D-HNU5, the calculated MDDFs also show, that the contributions of the nitrogen atoms are insignificant, and the hydrogen atoms are responsible for the major part of the interactions between guest molecules and host system (see Figure 4). Closer inspection of the contributions of the individual involved hydrogen atoms reveals, that in contrast to HEX-COF1 a larger number of distinct hydrogen atoms contributes to the total MDDF. While hydrogens 5 and 6 still show a dominant contribution, they can not be identified as the main modality of interaction between CO_2_ and the 3D-HNU5 material. In contrast, the dominating contribution originates from hydrogens 2 and 9, which can still be considered in vicinity of the azine group. In any case it seems plausible that these hydrogen atoms are still electronically under strong influence of the azine group, especially considering the conjugated nature of the entire linking unit.

Certainly, these different hydrogen contributions observed in case of HEX-COF1 and 3D-HNU5 involved in the interactions with CO_2_ guest molecules seems surprising, considering that they are composed of identical linker moieties. However, the observed deviation may be the result of their differences in structure and flexibility. In the three-dimensional 3D-HNU5, the linkers connect a single carbon atom forming the central unit and as such the linkers are arranged in a tetrahedral geometry with two linkers enclosing an angle of approx. 109.5°. This comparably large angle renders all linker hydrogen atoms well accessible. In contrast, in the planar HEX-COF1 the azine linkers connect benzene central moieties, with two linkers spanning an angle of 60° rendering the terminal hydrogen atoms (H_1_, H_3_, H_8_ and H_10_ in Figure 1A) significantly less exposed.

Although the polar azine groups seem to influence the CO_2_ adsorption in both systems, the experimentally determined maximum CO_2_ uptake capacities diverge. Consequently, it stands to reason, that there are more factors to be considered, one of which certainly is the pore size. To this effect, the publication introducing HEX-COF1 Alahakoon et al. (2016) suggests, that the reported, excellent CO_2_ adsorption capacity of 20.0 w% may, in addition to the favourable effect of the azine groups, be further enhanced by the small pore diameter of 1.1 nm. However, for 3D-HNU5, which contains the same azine linkers and is reported to have a similarly small pore diameter of 1.0 nm, the experimentally reported CO_2_ adsorption capacity of 12.3 w% is significantly decreased Alahakoon et al. (2016); Guan et al. (2019), *i.e.*, although both presented COFs display both features, the adsorption capacities diverge significantly. One possible explanation could be the fact that both measurements are conducted by two distinct research groups, which might reduce the ultimate comparability of the data sets. In addition, further governing influences such as the difference between the respective 2D and 3D frameworks can be expected to further influence the binding properties, e.g., the spatial proximity of azine units in HEX-COF1 display cooperative effects in CO_2_ binding compared to the isolated azine units in 3D-HNU5.

Considering, that the experimentally determined pore size distributions of both COF systems result from N_2_ adsorption isotherms at 77.15 K and the determination of the adsorption capacity was carried out at 273.15 K, in the following the temperature dependence of the pore size in both systems is investigated.

### 3.5 Pore size distribution

**FIGURE 7:**
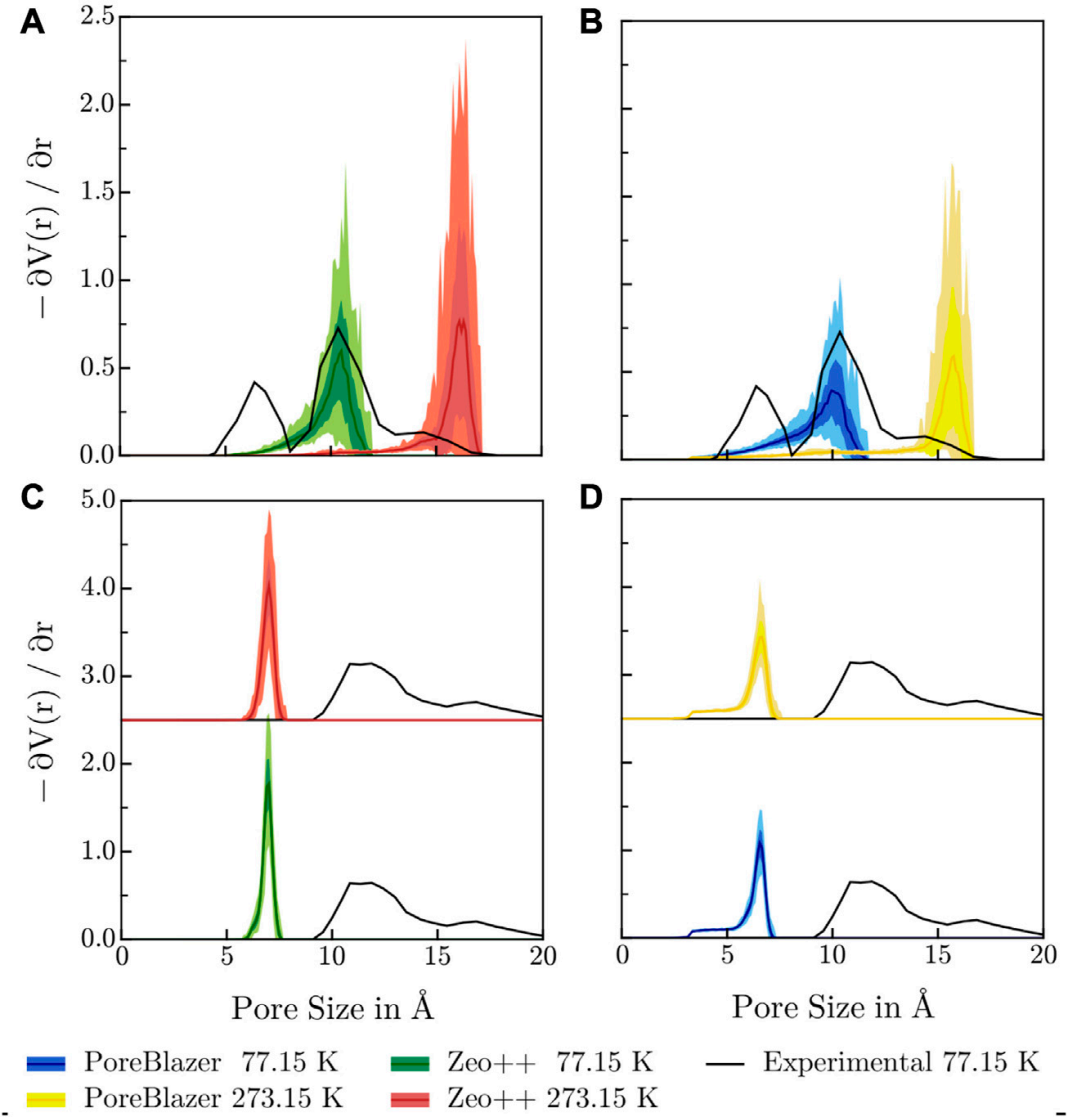
Comparison of the PSDs determined *via* the respective pore volume gradient as a function of pore radius, calculated with **(A, C)** Zeo++ and **(B, D)** PoreBlazer at 77.15 K and 273.15 K with the PSD determined from adsorption experiments at 77.15 K for **(A, B)** 3D-HNU5 and **(C, D)** HEX-COF1. The figure shows the calculated average *μ* enclosed by the interval *μ* ± *σ*, as well as the range of the y values for a given pore size [*y*_min_, *y*_max_] indicated via the color shading. For HEX-COF1, the PSDs at 273.15 K are shifted up, since the data at both temperatures are almost indistinguishable.

To further examine the quality of the simulation results and especially how well they correspond to experimental data, the associated pore size distributions (PSDs) were calculated using two different geometric methods. To generate a representative distribution, 100 geometries equally spaced in time were drawn from a 1.0 ns equilibrated simulation at the target temperature and the associated PSDs averaged. Figure 7 depicts a comparison of the PSDs for both investigated systems calculated *via* both geometrical methods at 77.15 K and 273.15 K with the experimental reference data at 77.15 K. The experimental PSDs are only presented up to a pore size of 20 Å, because peaks larger than that do necessarily arise from mesoscopic effects that can not be represented in modestly sized unit cells.

It can be noted that for 3D-HNU5 at 77.15 K the agreement between the experimental and calculated PSD is remarkably good, pointing towards an average pore radius of approx. 10 Å. At 273 K the maximum in the calculated PSD is shifted to approx. 16 Å which reflects the temperature dependent structural deformation mentioned earlier (*cf.* Section 3.3). Consequently, this finding suggests, that the experimental PSD may not reflect the properties of the material under the conditions the adsorption experiments were carried out being 273.15 K. Furthermore, this challenges any rationalizations of the experimentally observed adsorption behaviour based on the PSD measurements. The fact, that the experimental PSD shows a second peak at approx. 6 Å with no calculated complement may be due to experimental or methodological shortcomings or inherent limitations of the MD framework. In this context, the second peak visible in the experimental PSD might stem from an irregular mesoscopic structure, *i.e.*, parts exist *e.g.*, in a 3-fold interpenetrated or non-interpenetrated diamond topology, instead of the prevalent 2-fold interpenetrated one. Unfortunately, these effects can not be represented in modestly sized simulation cells.

Furthermore, for the estimation of the PSD from the measured adsorption isotherms non-local DFT (NLDFT) was used in conjunction with a kernel based on the infinite slit-pore model geometry. To this effect, the bipartite nature of the experimental PSD of 3D-HNU5 may be an artifact from the NLDFT based estimation, which have been reported in the range <1nm and have been attributed *inter alia* to the homogeneous surface model used Cornette et al. (2020). As shown by Kupgan et al. Kupgan et al. (2017), even for computationally simulated adsorption isotherms, consequently excluding experimental factors, pore size distributions based on the slit-pore model show alarming deviations from the associated geometrical PSDs for a wide range of microporous chemical systems, questioning the application of the former without careful validation.

For HEX-COF1, the calculated PSDs at 77.15 K and 273.15 K are almost indistinguishable and show a single maximum at approx. 7 Å, whereas in the experimentally determined PSD approx. 11 Å can be identified as most prevalent pore size. Both software packages, PoreBlazer and Zeo++, conceptually determine the maximum pore size by calculating the largest sphere, that a given pore can accommodate. Thus, it seems plausible that for pore geometries with triangular cross-section, as present in HEX-COF1, the obtained value of approx. 7 Å is slightly lower compared to a rough estimate based on the side length of the cross-sectional triangle^2^, corrected for van-der-Waals radii, amounting to approx. 12 Å. Further, the experimental PSD for HEX-COF1 was estimated from the adsorption isotherms *via* the infinite slit-pore model, and unfortunately it remains unclear how reliably the latter can be applied to this uncommon pore shape. Further, in the experimental adsorption measurements for both HEX-COF1 and 3D-HNU5, N_2_ at 77 K was used as probing gas, which is known to exhibit specific interactions with polar functional groups in the sorbent due to its quadrupole moment. Consequently, IUPAC recommends Ar at 87 K for the characterization of micropores in sorbents that contain polar surface moieties Thommes et al. (2015), *e.g.*, MOFs as well as certain COF systems.

Upon comparison of the calculated PSDs, the temperature dependent deformation of 3D-HNU5 is clearly visible, while in the rigid HEX-COF1 structure the temperature has little influence on the calculated pore size. Further, it is immediately obvious, that the individual PSDs calculated from frames of the simulation trajectory show hardly any variation in the case of HEX-COF1. For 3D-HNU5, however, the averaged PSDs have a large standard deviation, which again highlights the pronounced structural flexibility in this case compared to the rigid HEX-COF1.

### 3.6 Diffusion analysis

**FIGURE 8:**
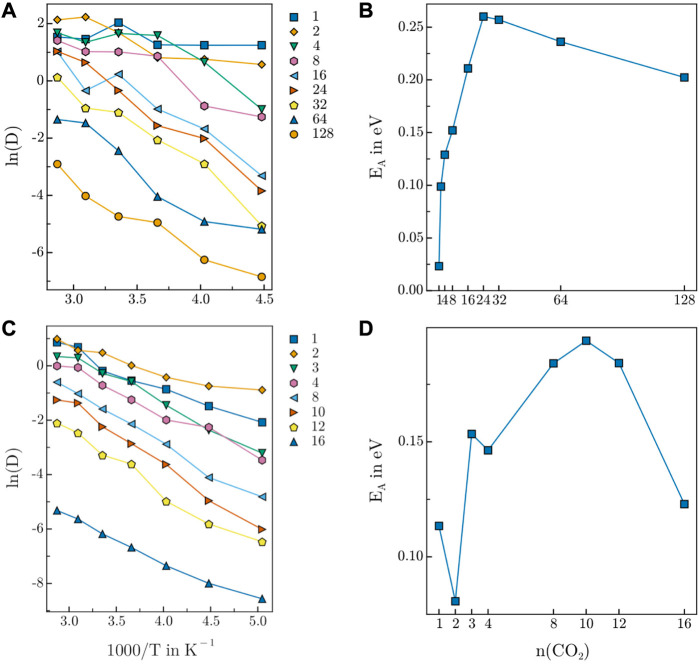
Arrhenius plot of CO_2_ diffusion in **(A)** 3D-HNU5 and **(B)** HEX-COF1. Activation energy of CO_2_ diffusion in **(C)** 3D-HNU5 and **(D)** HEX-COF1. The CO_2_ count in the legend of **(C)** and the label on the x-axis of **(D)** refers to the CO_2_ content per pore.

The diffusion coefficients of the guest molecules have been determined from the simulation trajectory *via* the Einstein-Smoluchowski relation considering both variations in concentration and temperature. The respective Arrhenius plots for HEX-COF1 and 3D-HNU5 are depicted in Figure 8A, C.

Generally, both Arrhenius plots show the expected behaviour of an exponential decrease of the diffusion coefficients relative to the inverse temperature. Moreover, a decrease of the diffusivity upon increasing concentration of guest molecules is observed. In general, the fact that the Arrhenius plots for 3D-HNU5 are less smooth is likely related to the circumstance, that the *NPT* ensemble was sampled as opposed to the *NVT* ensemble in the case of HEX-COF1. Further, for 3D-HNU5 two outliers are observed for 8 CO_2_ molecules at 273.15 K and 16 CO_2_ molecules at 323.15 K. However, also by drastically increasing the equilibration time, these data points did not change significantly. Additionally, the sampling quality is inherently worse for lower numbers of present guest molecules as expected and consequently the data for 1, 2, and 4 CO_2_ molecules is not clearly separated.

In the case of HEX-COF1, the activation energies for 1 and 3 CO_2_ molecules per pore are too large compared to the remaining data points. The fact that these data points are the only ones corresponding to systems with an odd number of guest molecules per pore suggests the presence of a particular type of interaction in the guest-host system, that is favourable only for an odd number of CO_2_ molecules. The activation energies *E*_*A*_ determined for both examined systems as a function of CO_2_ content (Figures 8B, D) show a sharp initial increase up to certain loading followed by a gradual decrease.

At low concentrations, each CO_2_ molecule has a multitude of vacant binding sites nearby, which can be expected to encourage diffusion. The initial increase in activation energy of diffusion also suggests, that the interaction of a single CO_2_ molecule further favours the adsorption of additional guest molecules. Mechanistically, this may be mediated *via* intramolecular charge transfer and although the NNP ANI-2x does not explicitly take such varying partial charges into account, one may assume, that due to the training to data produced with the DFT functional ωB97X this effect is implicitly reflected.

As more CO_2_ molecules are introduced, the number of diffusion facilitating vacant sites declines which is reflected in an increase of the activation energy of diffusion. The behaviour of increased favourability is observed as long as free interaction sites are available. Adding further CO_2_ molecules after all interaction sites are occupied, leaves free CO_2_ molecules, that diffuse very easily and compete with bound guest molecules for the available binding positions, which is clearly reflected by the decrease of the activation energy of diffusion for larger CO_2_ concentrations, resulting in the exhibition of a maximum in Figures 8B, D.

The maxima of the activation energy in Figures 8B, D are observed at 24 CO_2_ molecules (15.6 w%) and 10 CO_2_ molecules per pore (21.2 w%) for 3D-HNU5 and HEX-COF1, respectively. Interestingly, this data is in very good agreement with the empirically determined CO_2_ uptake capacity of 12.3 w% CO_2_ and 20.0 w% CO_2_, obtained from the measured adsorption isotherms at pressures up to 1.0 bar and 1.0 atm respectively, supporting the findings of this work.

## 4. Conclusion

To conclude, this work was able to demonstrate, that the NNP ANI-2x is suitable to adequately describe macromolecular structures such as COFs, as well as their interaction with CO_2_ guest molecules although these systems were not explicitly considered in the training. However, noteworthy limitations include the inability to model macroscopic effects entailed by the simulation on a molecular scale as well as the difficulties to quantitatively reflect the CO_2_-COF interaction energy with ANI-2x, observed in the case of HEX-COF1. In consideration of the comparison of the PSDs, it remains unclear whether the observed deviations can be ascribed to limitations in the experimental or theoretical protocols. Nevertheless, it can be concluded that the ANI-2x NNP facilitates the calculational treatment at approx. DFT accuracy and force field cost. Essentially, the key innovation presented in this study is the application of a DFT-based neural network approach with a suitable MD simulation protocol enabling the treatment of a (comparably) large system for very long simulation times providing access to correlated properties in the long-time limit which in this case is the diffusion coefficient. In particular, the outstanding efficiency is illustrated by the extensive overall simulation time amounting to more than 0.4 µs, which is impossible to achieve with DFT methods.

Further this project developed a workflow, that facilitates the estimation of the maximum CO_2_ adsorption capacity, which reproduced the experimental results astonishingly well. Also, this method is easily generalizable to other host systems as well as different guest molecules and as such can be used to predict maximum adsorption capacities of a variety of systems and as such guide further experimental work.

Additionally, this work illustrates, that MDDFs can be a useful tool in understanding the nature of interactions in host-gas systems, revealing similarities but also major differences between HEX-COF1 and 3D-HNU5 in host-CO_2_ interactions, the latter of which are likely attributable to the differences in structure.

## Data Availability

The raw data supporting the conclusion of this article will be made available by the authors, without undue reservation.

## Footnotes

1 The number of significant figures was chosen in accordance with the obtained standard deviation.

2 The diameter of a circle inscribed in a triangle is $d=2\cdot \sqrt{\frac{\left(s-a\right)\left(s-b\right)\left(s-c\right)}{s}}$ , where $s=\frac{a+b+c}{2}$ . Thus for *a* = *b* = *c* = 12: *d* = 6.93.

## Abbreviations

DFT, density functional theory; IPCC, Intergovernmental Panel on Climate Change; QM, quantum mechanical; MM, molecular mechanical; NNP, neural network potential; ANI, Accurate Neural network engine for Molecular Energies, ANAKIN-ME; COF, covalent organic framework; MOF, metal organic framework; MD, molecular dynamics; ML, machine learning; AEV, atomic environment vector; DFTB, density functional tight binding; *NVT* ensemble, canonical ensemble; *NPT* ensemble, isothermal-isobaric ensemble; MDDF, minimum distance distribution function; RDF, radial distribution function; PSD, pore size distribution; NLDFT, non-local DFT.
