# Supplementary material for: Storage and diffusion of CO2 in covalent organic frameworks—A neural network-based molecular dynamics simulation approach
Source: Front Chem. 2023 Mar 9;11:1100210. doi: 10.3389/fchem.2023.1100210 (PMC10033539; doi:10.3389/fchem.2023.1100210)
Supplement: Supplementary file 1 [file Presentation1.pdf]

Supplementary Material

1.1 Space Group of HEX-COF1

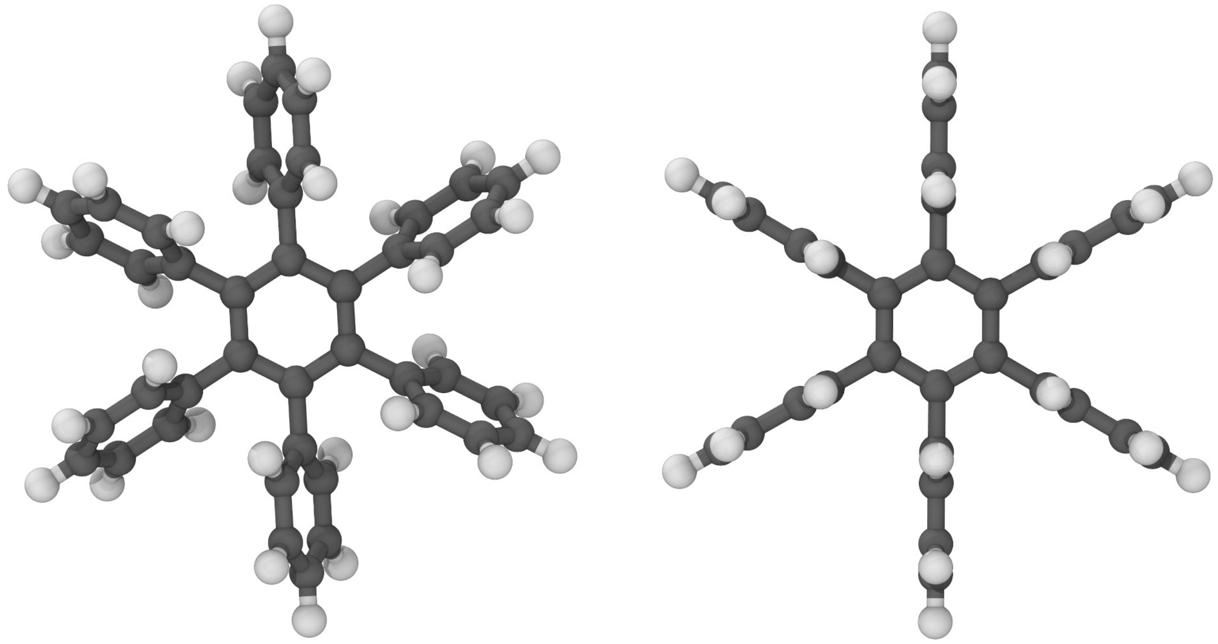

Figure S1. Comparison of biphenyl angle in hexaphenylbenzene, conformation corresponding to HEX-COF1 in P6 space group (left) and P6/m (right). In contrast to the latter, showing a biphenyl angle of exactly $90°$, the biphenyl angle in space group P6 ammounts to $68.3°$. In addition, the tilted conformer shows the lower energy by $8.4\,\mathrm{kJ \cdot mol^{-1}}$

1.2 Artificial CO_2-CO_2 interactions across the periodic boundary

In order to confirm, that the chosen system sizes for 3D-HNU5 (unit cell) and HEX-COF1 (1x1x3) are sufficient to avoid possible artificial CO_2-CO_2 interactions across the periodic boundary, the interaction energy was calculated according to Eq. (S1), where E_{int}, $E_{CO_2}^{PBC}$ and $E_{CO_2}^{vac}$ correspond to the CO_2-CO_2 interaction across the periodic boundary, the total energy of a single CO_2 molecule in an empty cell with the same size as the COF cell and the energy of a single CO_2 molecule *in vacuo*, respectively.

$$E_{int} = E_{CO_2}^{PBC} - E_{CO_2}^{vac} \tag{S1}$$

With DFTB, this resulted in interaction energies of -0.0058 kJ/mol and -0.0037 kJ/mol for HEX-COF1 and 3D-HNU5, respectively, which confirms, that for the chosen cell sizes CO_2-CO_2 interactions across the periodic boundary are insignificant and the cell sizes are deemed adequate.

1.3 Simulation Protocol

Table S1. Target temperatures for 3D-HNU5 and HEX-COF1

	T / K
3D-HNU5	223.15
	248.15
	273.15
	298.15
	323.15
	348.15
HEX-COF1	198.15
	223.15
	248.15
	273.15
	298.15
	323.15
	348.15

1.4 Pore Size Distribution

Table S2. Pore limiting diameter (PLD), largest cavity diameter (LCD) and mean pore diameter (MPD) for HEX-COF1 and 3D-HNU5, calculated via the Zeo++ and PoreBlazer software. The MPD was determined as weighted average of the PSD.

		3D-HNU5		HEX-COF1	
T / K		77.15	273.15	77.15	273.15
PLD	PoreBlazer	7.80	12.7	5.60	5.73
	Zeo++	8.00	13.0	5.93	6.03
LCD	PoreBlazer	11.1	16.2	7.04	7.15
	Zeo++	11.3	16.6	7.36	7.44
MPD	PoreBlazer	8.90	13.6	5.98	6.02
	Zeo++	9.85	15.4	6.90	6.93
